# Improving long-term postoperative survival in a porcine cardiac valve surgery model utilizing cardiopulmonary bypass via left thoracotomy: a single-center experience sharing insights

**DOI:** 10.3389/fcvm.2024.1427653

**Published:** 2025-01-08

**Authors:** Qingping Xia, Yong Cao, Jialuan Li, Jie Jiang, Xuan Lu, Li Deng

**Affiliations:** ^1^ Department of Science and Education, Gaozhou People’s Hospital, Gaozhou, Guangdong, China; ^2^ Department of Cardiovascular Surgery, The People’s Hospital of Gaozhou, Gaozhou, Guangdong, China; ^3^ Silver Snake (Guangzhou) Medical Science and Technique Co., Ltd., Guangzhou, Guangdong, China; ^4^ Department of Cardiovascular Surgery, Gaozhou People’s Hospital, Gaozhou, Guangdong, China

**Keywords:** porcine cardiac valve surgery, cardiopulmonary bypass, left thoracotomy, postoperative survival, follow -up

## Abstract

**Objective:**

The objective of this study was to improve long-term postoperative survival in a porcine cardiac valve surgery model by utilizing cardiopulmonary bypass (CPB) via left thoracotomy. The study aimed to share refined techniques and insights accumulated over years at a single-center animal clinical trial facility.

**Method:**

A total of 196 Chinese Large White pigs weighing between 60 and 75 kg were used in the study. All animals underwent cardiac valve surgeries via left thoracotomy with CPB. Surgical techniques included mitral valve replacement, mitral valve repair, aortic valve replacement, OZAKI procedure, ascending aorta replacement, and left ventricular assist device implantation. Anesthesia and CPB protocols were optimized to minimize stress and complications. Postoperative care was standardized to enhance recovery and survival.

**Result:**

All 196 pigs survived the surgical procedures, with no deaths reported. The mean surgical duration was 168.55 ± 38.75 min, CPB time was 114.89 ± 32.11 min, and aortic cross-clamp time was 76.75 ± 21.33 min. Automatic heart resumption occurred in 63.8% of pigs, while the remainder required electrical defibrillation or cardiac massage. The postoperative mechanical ventilation time was 2.44 ± 0.58 min, and the average drainage volume at 2 h postoperatively was 27.50 ± 9.70 ml. There were no cases of postoperative hemorrhage complications or blood transfusions, and surgical site infections occurred in only 1.5% of pigs.

**Conclusion:**

The surgical approach utilizing left thoracotomy with CPB has proven effective in significantly enhancing long-term survival rates in porcine heart surgeries. The refined techniques and standardized operational procedures described in this study offer valuable insights for researchers aiming to improve the success of porcine heart valve surgical models. However, due to differences in animal anatomy, the applicability of this surgical approach to other animal models still requires further exploration.

## 1 Introduction

Pigs are frequently used in medical research studies due to their anatomical and physiological similarities to humans (1, 2). The porcine heart surgical model is particularly crucial for research, clinical skill training, and surgical education (3, 4). However, when using large animal surgical models like pigs, several obstacles come to the fore. Financial limitations often restrict the number of experiments that can be conducted, and space constraints may affect the animals' living conditions during the study period. Technically, the absence of standardized surgical protocols poses a significant challenge. Without proper expertise and perioperative management techniques, not only are the economic costs of these experiments inflated, but also the failure rates increase.

Among the various types of pigs used in experiments, the small—sized Bama pigs are commonly considered. Unfortunately, their postoperative survival rates are so low that they present a major hurdle for long—term survival model—based research, clinical observations, and follow—up studies. This is mainly because these pigs have unique physiological and anatomical features that make them more vulnerable during and after surgery (5).

Our team initially focused on performing valve surgeries in pigs using a median sternotomy approach. In this process, we encountered two major issues. Firstly, due to the specific anatomical structure of pigs, the mitral and aortic valves were difficult to expose adequately through this technique. Secondly, median sternotomy involves splitting the sternum, which causes severe trauma to the pigs. This trauma not only affects the immediate postoperative recovery but also leads to difficult sternal healing. As a result, the combination of these factors significantly contributes to a high postoperative mortality rate.

To address these problems, we turned our attention to Chinese Large White pigs. Through extensive research, we explored the valve surgical experience with cardiopulmonary bypass (CPB) in these pigs. This new approach has shown promising outcomes. In this article, we, from the Silver Snake Animal Clinical Trial Center, aim to share the refined techniques and insights accumulated over years. By doing so, we hope to assist researchers in reducing their learning curve and enhancing the success rate of porcine heart valve surgical models.

## 2 Methods

### 2.1 Experimental animals

A total of 196 Chinese Large White pigs, including 109 males, weighing between 60 and 75 kg, were sourced from Jiangxi Silver Snake Experimental Animal Breeding Farm. This study was granted ethical approval (Approval Number: SS-2023-GZ1) by the Experimental Animal Welfare and Ethics Committee of Guangzhou Silver Snake Clinical Trial Center.

### 2.2 Instruments and equipment

(1) Cardiopulmonary bypass machine and water tank: Stöckert S5 model (SorinGroup, Germany); (2) Membrane oxygenator: adult-sized [Dongguan Kewei Medical Instrument Co., Ltd. (China)] or integrated membrane oxygenator (Terumo, Japan); (3) Animal anesthesia machine: Dräger-Trio, Drägerwerk (AG & Co. K GaA, Germany); (4) ACT analyzer: ACT II model (Medtronic Inc., USA).

### 2.3 Anesthesia and CPB

#### 2.3.1 Anesthesia protocol

Animals underwent a 12-hour fasting period before surgery. They received premedication with atropine sulphate and Zoletil. They were then anesthetized and ventilated mechanically via endotracheal intubation. Ventilation parameters were adjusted for optimal respiratory function. Neuromuscular blocking agents were not administered. Anesthesia was maintained through intravenous infusions. Vital signs, including ECG, respiratory rate, end-tidal carbon dioxide (ETCO_2_), and blood oxygen saturation, were continuously monitored. Temperature probes were inserted into the nasopharynx and rectum. Preoperative and intraoperative echocardiography were conducted. ECG electrodes were affixed to the limbs. Jugular venous and femoral arterial catheters were inserted for volume monitoring, fluid replacement, and continuous invasive blood pressure monitoring.

#### 2.3.2 Technique of cardiopulmonary bypass

The priming fluid consisted of 500 ml of Hydroxyethyl Starch130/0.4 in Sodium Chloride Injection and 1,000 ml of Ringer's solution mixed in a 1:2 ratio (500 ml to 1,000 ml). Intraoperatively, the activated clotting time (ACT) was kept between 500 and 600 s; blood flow at 40–60 ml/kg; and mean arterial pressure maintained within the range of 60–80 mmHg. Adjustments during the operation were based on blood gas values, with vasoactive medications titrated based on hemodynamic parameters, and autologous blood transfusion devices were employed throughout the procedure.

### 2.4 Surgical technique

#### 2.4.1 Positioning and incision

**Figure 1:**
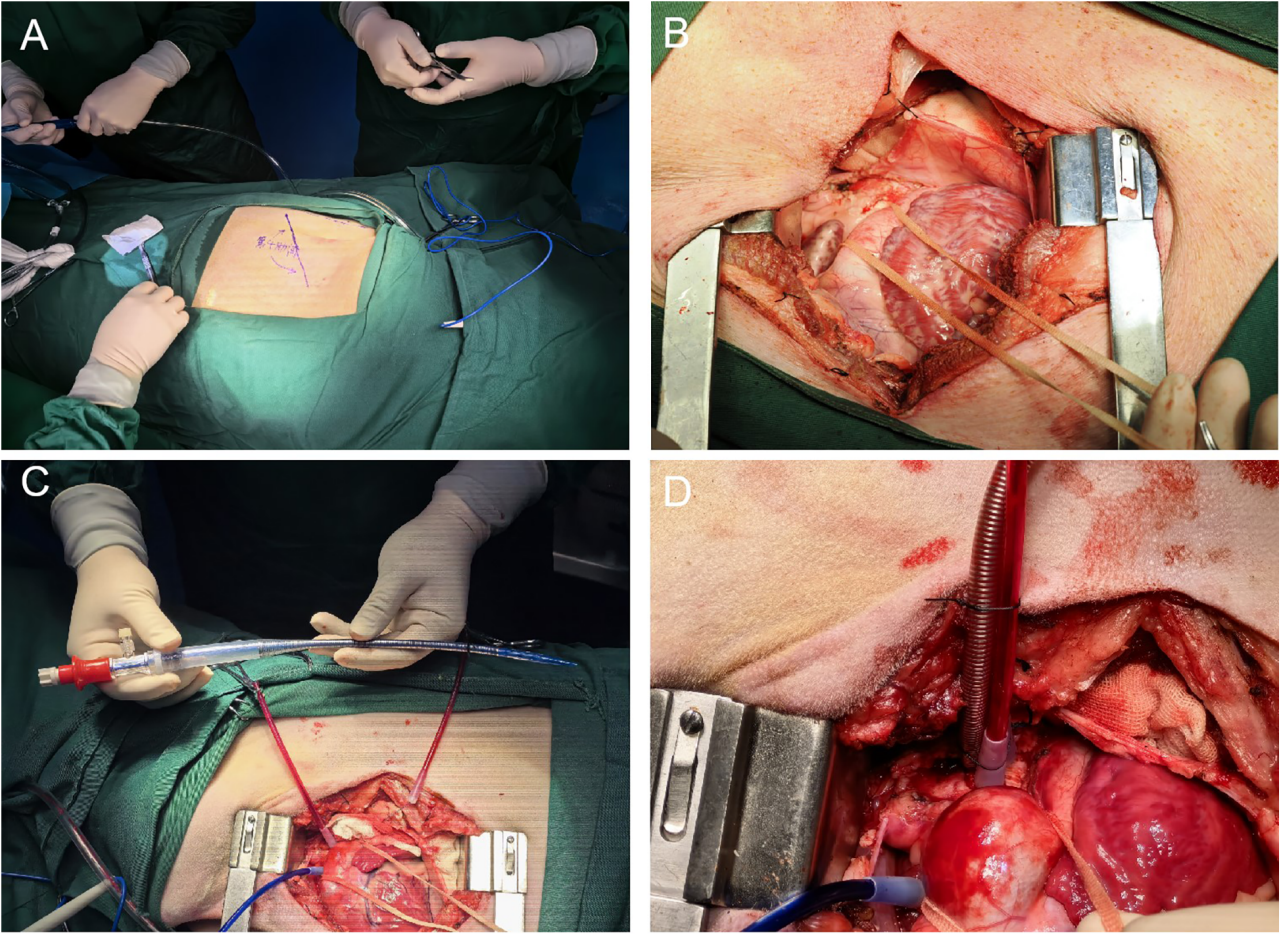
**(A)** Illustrates the positioning of the pig in the right lateral decubitus position with its limbs immobilized to the surgical table, and indicates the location of the surgical approach on the body surface corresponding to the fourth intercostal space on the left side, below the root of the left forelimb. **(B)** Depicts the left thoracotomy being carried out through the fourth intercostal space on the pig's chest, showing the sequential dissection through the skin, subcutaneous adipose tissue, and muscle layers to access the thoracic cavity. **(C)** Displays the aortic cannula used during the establishment of cardiopulmonary bypass (CPB), highlighting its characteristics and position in the surgical setup. **(D)** Demonstrates the successful insertion of the ascending aortic cannula into the aorta using the guidewire-assisted technique, with details on the insertion process and the securing of the cannula.

The pig was positioned in the right lateral decubitus position, with its limbs immobilized to the surgical table using ropes. The surgical site was sterilized and draped. An incision approximately 15–20 cm in length was made at the fourth intercostal space on the left side, corresponding to the area below the root of the left forelimb. Sequential dissection through the skin, subcutaneous adipose tissue, and muscle layers was performed to access the thoracic cavity (Figure 1A,B).

#### 2.4.2 The process of establishing ascending aorta cannulation with guidewire assistance

The pericardium of the pig was incised to expose the ascending aorta and aortic arch.Excess adipose tissue was excised, and a double-pouch suture was placed at the aortic arch using 4-0 Surgipro II polypropylene suture. An empty spacer was inserted, followed by placement of a suture applicator for subsequet use. After confirmation of an ACT time exceeding 480 s, a stiff guidewire was inserted through a puncture made at the center of the pouch using a puncture needle. The needle was withdrawn, and an 18F femoral artery cannula was inserted along the guidewire to a depth of approximately 5 cm. The suture applicator was tightened, and the cannula was secured with a 1–0 silk tie (Figure 1C,D).

#### 2.4.3 Venous cannulation with cavoatrial cannula

**Figure 2:**
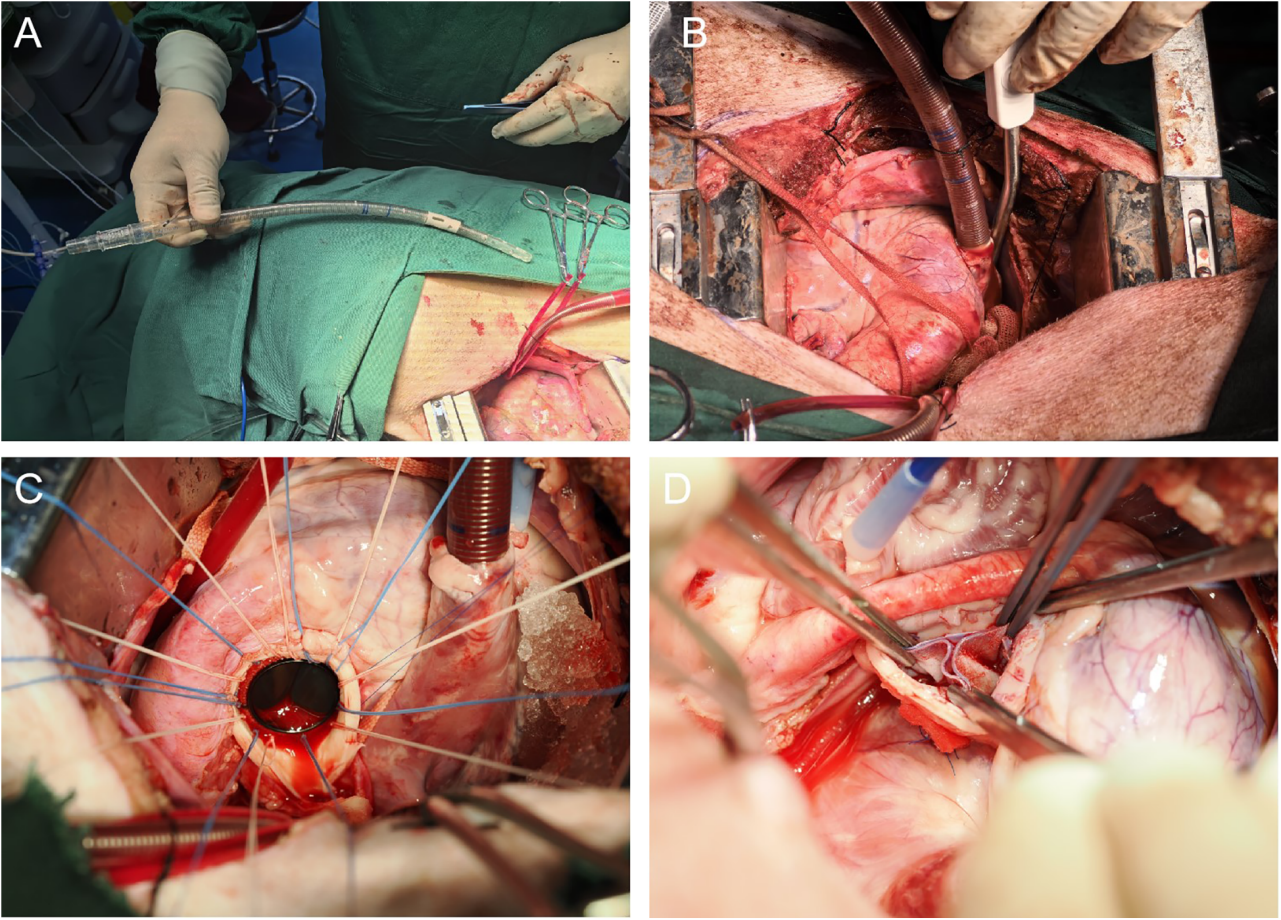
**(A)** Illustrates the process of performing right atrial venous drainage using an atrial—caval drainage cannula. This shows how the cannula is used in place of separate superior and inferior vena cava cannulas, emphasizing its role in the surgical technique for venous drainage. **(B)** Depicts the successful insertion of the atrial—caval tube into the right atrium. It shows the proper placement and insertion depth of the tube, which is crucial for establishing effective venous drainage during the surgical procedure. **(C)** The image depicts the process of mitral valve replacement, in which a mechanical valve prosthesis has been implanted using a horizontal mattress suture with 2-0 Ticron sutures. **(D)** The image highlights a key step in the OZAKI procedure, where the three leaflets of the aortic valve are reconstructed using a bovine pericardial patch.

A atrial clamp was used to grasp a portion of the right atrial appendage. A pouch was created at the appendage using 4-0 Surgipro II polypropylene suture, and a suture applicator with an empty spacer was inserted for later use. The center point of the pouch was cut using a sharp knife, and a 28–32F venous cannula was inserted. With guiding the cannula into the inferior vena cava with the left hand by palpating the opening, the cannula was advanced to the appropriate depth. The suture applicator was tightened, and the cannula was secured with a 1-0 silk tie (Figure 2A,B).

#### 2.4.4 Administration of cardioplegic solution

After the initiation of CPB, the adequacy of venous drainage through the cavoatrial cannula was confirmed. Adequate drainage was evidenced by the collapse of the pulmonary artery, facilitating the exposure of the ascending aorta. The purse-string suture was meticulously sewn onto the mid-portion of the ascending aorta utilizing a 4-0 Surgipro II polypropylene suture reinforced with a felt pledget. The completed purse-string suture then enabled the insertion of a cardioplegic solution infusion needle. The suture applicator was tightened, and the purse-string suture was secured with a 1-0 silk tie. The myocardial protection perfusion device was connected, and intraoperatively, DelNido cardioplegic solution was employed for myocardial protection with a perfusion rate of 30 ml/kg/min for a duration of 5–8 min. After completing the perfusion, gauze and ice slurry were placed around the pericardium.

#### 2.4.5 Description of surgical procedures

##### 2.4.5.1 Mitral valve replacement

An incision was made in the middle portion of the left atrial appendage, and a left atrial cannula was inserted into the inferior left pulmonary vein for venous drainage. After achieving a clear surgical field, the anterior leaflet and chordae tendineae of the mitral valve were excised, while the posterior leaflet was preserved. A valve sizer was utilized to determine the appropriate valve size. A horizontal mattress suture with 2-0 Ticron suture reinforced with felt pledgets was placed along the mitral annulus for 12–14 stitches to secure the new valve in place. After tying the sutures, the left atrium was closed with a 5-0 Surgipro II polypropylene suture. Following the removal of any air from the heart chambers (de-airing), the heart was reperfused (Figure 2C).

##### 2.4.5.2 Mitral valve repair

An incision was made in the middle portion of the left atrial appendage, and a left atrial cannula was inserted into the inferior aspect left pulmonary vein. With a clear surgical field, 8–12 stitches were placed along the mitral annulus using 2-0 Ticron polyester suture. A C-shaped annuloplasty ring was implanted and secured with sutures. After confirming the absence of valve regurgitation by injecting water into the left ventricle, the left atrium was closed with 5-0 Surgipro II polypropylene suture. Following de-airing, the heart was reperfused.

##### 2.4.5.3 Aortic valve replacement

The ascending aorta was cut transversely to expose the aortic valve. The three cusps were excised along the annulus, and a valve sizer was used to determine the appropriate valve size. A horizontal mattress suture with 2-0 Ticron suture reinforced with felt pledgets was placed for 12–14 stitches. After tying the sutures, the ascending aorta was closed with 5-0 Surgipro II polypropylene suture. Following de-airing, the heart was reperfused.

##### 2.4.5.4 OZAKI procedure

The ascending aorta was cut transversely to expose the aortic valve. The three cu were excised along the annulus, and OZAKI valve sizers were used to measure the appropriate leaflet sizes. Continuous suturing of the three leaflets was performed using 5-0 Surgipro II polypropylene suture according to the OZAKI method, with reinforcement at the commissures using 4-0 Surgipro II polypropylene sutures. The sutures were passed through the aortic wall and secured with felt pledges. After confirming the proper alignment of the leaflets, the ascending aorta was closed with 5-0 Surgipro II polypropylene suture. Following de-airing, the heart was reperfused (Figure 2D).

##### 2.4.5.5 Ascending aorta replacement

A segment of the ascending aorta was transversely excised. An artificial vessel was trimmed to the appropriate length and anastomosed to both ends of the native aorta using a continuous suture of 5-0 Surgipro II polypropylene. After de-airing, the clamps were released, and the heart was reperfused. The anastomosis was inspected for bleeding.

##### 2.4.5.6 Left ventricular assist device implantation

A horizontal pledgeted suture with 3-0 Surgipro II polypropylene suture reinforced with felt pledgets was placed for 8–12 stitches at the apex of the heart. The sutures were passed through the base of the assist device and tied to secure it in place. A hole approximately 10 mm in diameter was created in the base using a dedicated punch and surrounding myocardial tissue was removed. The assist device was then inserted and secured to the base. A round hole approximately 8 mm in diameter was created in the aortic root using an aortic punch, and an end-to-side anastomosis between the artificial vessel and the aorta was performed using 5-0 Surgipro II polypropylene suture to connect the device to the aorta. Following de-airing, the heart was reperfused.

### 2.5 Additional steps

After completing the intracardiac procedures, the heart was thoroughly de-aired, and the ascending aorta was re-exposed. Once satisfactory restoration of heart rhythm and hemodynamic stability was achieved, the CPB machine was gradually discontinued. The cannula was removed, and heparin was neutralized with protamine sulfate injection. Bleeding was meticulously achieved, and a pleural drainage tube was inserted before closing the chest layer by layer. A suction bulb was attached for drainage lasting 1–3 days. The animal was then transferred to the recovery table for resuscitation. The animal was then transferred to the recovery table for resuscitation. Once the animal fully regained consciousness, the endotracheal tube was removed, and the animal was placed in an individual cage for feeding. Femoral arterial invasive blood pressure monitoring was maintained for at least 24 h and discontinued once hemodynamic stability was achieved. Central venous access was maintained for 1–3 days and withdrawn once normal feeding patterns resumed. Food was withheld on the day of surgery, and normal feeding resumed after 24 h, with meals adjusted according to the pig's condition, favoring smaller, more frequent meals. Postoperatively, the animal received 800,000 units of penicillin G intramuscularly twice daily for 7 days. Routine wound disinfection was performed to prevent infection. After reaching the expected post-operative follow-up period, these pigs were euthanized.

### 2.6 Statistical analysis

All the data were analyzed using SPSS software. The continuous variables were presented as mean ± standard deviation or as median with interquartile range. Continuous variables were compared using a standard one-way analysis of variance (ANOVA) if they met the assumptions of normality and homogeneity of variances. A *P*-value < 0.05 was considered to be statistically significant.

## 3 Results

A total of 196 pigs were included in the study, all of which survived, and there were no deaths.The longest survival time was 24 months, reaching the maximum survival duration according to the experimental design. The mean surgical duration was 168.55 ± 38.75 min, with a CPB time of 114.89 ± 32.11 min and an aortic cross-clamp time of 76.75 ± 21.33 min. There were 0 cases of off-pump surgery. Automatic heart resumption occurred in 125 pigs (63.8%), while 71 pigs (36.2%) required electrical defibrillation or cardiac massage for heartbeat restoration. The postoperative mechanical ventilation time was 2.44 ± 0.58 min. The average drainage volume at 2 h postoperatively was 27.50 ± 9.70 ml. There were 0 cases of postoperative hemorrhage complications or blood transfusions. Surgical site infections occurred in 3 pigs (1.5%). Detailed information is presented in Table 1.

**Table 1:** Clinical characteristics and data of the patients(x¯ ± s) (*n* = 196).

Variables	Data
Preoperative characteristics	
Age (M)	6.81 ± 0.91
Male (*N*/%)	109（56%）
Weight (Kg)	67.56 ± 3.83
Intraoperative characteristics	
MVP (*N*/%)	31（15.8%）
MVR (*N*/%)	66（33.7%）
AVR (*N*/%)	8（4%）
Ozaki (*N*/%)	8（4%）
AAR (*N*/%)	35（18%）
DAR' (*N*/%)	3（1.5%）
Left heart assist device (*N*/%)	45（22.9%）
Superior and inferior vena cava cannulation (*N*/%)	2（1%）
Right Atrial Cannulation (*N*/%)	194（99%）
Off-pump Surgery (*N*/%)	0
On-pump surgery (*N*/%)	196（100%）
The average operating time	168.55 ± 38.75
CPB time (min)	114.89 ± 32.11
Cross-clamp time (min)	76.75 ± 21.33
ROSC（%）	125（63.8%）
Postoperative characteristics	
Ventilation time (h)	2.44 ± 0.58
Recovery feeding time	41.94 ± 4.61
Average drainage（ml）	54.09 ± 23.3
Infection complication (%)	
Incision infection	3（1.5%）
Other infection	0
Mean survival time (M)	9 (3,24)
Mortality in center (*N*/%)	0

MVP, mitral vavuloplasty; MVR, mitral valve replacement; AVR, aortic valve replacement; AAR, ascending aorta replacement; DAR, descending aorta replacement; LVAD, left ventricular assist device; ROSC, the rate of spontaneous return of spontaneous circulation.

## 4 Discussion

Pigs are widely recognized as the optimal choice for animal trials in various cardiac surgeries due to their close anatomical resemblance to the human heart (6, 7). The selection of Chinese Large White pigs for our study is based on their body size, which closely mirrors that of adults, making them particularly suitable for preclinical trials of cardiovascular products. Their remarkable tolerance to diseases and relatively low mortality rate even after significant surgical trauma further highlight their value as an experimental model in cardiovascular research (8, 9).

However, it should be noted that despite the similarities, pigs and humans, as distinct species, possess certain morphological differences in their thoracic cavities and hearts. Previous studies have shown that these differences prevent the direct replication of human open—heart surgical models onto pig models (8, 10). For instance, in our initial attempts to perform valve surgeries using a median sternotomy approach, we encountered significant difficulties in achieving optimal exposure of the valve positions, which in turn hindered the successful completion of various valve procedures. In contrast, the intercostal thoracotomy approach we subsequently explored not only enabled the successful establishment of a cardiopulmonary bypass model but also provided ideal exposure for the aortic and mitral valves. Nevertheless, for procedures such as coronary artery bypass grafting or heart transplantation, the median sternotomy approach may still be a viable option (11).

This shift to the intercostal thoracotomy approach offers a significant advantage in terms of reduced trauma, which is conducive to the long—term survival of the animals post—surgery. As far as we know, no articles similar to our valvular surgery model have been found published so far, thus highlighting the novelty of our work.

In addition to the surgical approach, we have also optimized the anesthesia and CPB protocols. We deliberately avoided the use of muscle relaxants during anesthesia. This decision is based on previous research which shows that this method promotes the rapid awakening and extubation of animals after surgery, without any respiratory—related postoperative complications (12, 13). Moreover, compared to humans, pigs have a smaller effective circulating volume and greater vascular endothelial permeability, which predispose them to edema (14, 15). To address this issue, we adopted a priming solution with a crystalloid—to—colloid ratio of 1:2, as suggested by relevant physiological studies on porcine models (16). Considering the complexity of cross—matching due to the existence of 15 blood types in pigs, we took measures to minimize blood dilution (17). Specifically, we recommend shortening the circulation time and using an integrated oxygenator. Additionally, intraoperative autologous blood transfusion techniques were employed to reduce blood loss. Generally, blood transfusion is not required when hemoglobin levels are above 6 g. Given the higher body temperature of pigs, the priming solution was preheated to 32°C, and the intraoperative temperature was maintained between 30°C and 34°C, followed by gradual rewarming to 38°C after the procedure. These comprehensive measures have been shown to significantly reduce CPB—related complications and accelerate the recovery of pigs (18).

Surgical technique modifications in pig heart surgeries are of utmost importance. Firstly, due to the short and fragile ascending aorta in pigs, guidewire—assisted ascending aortic cannulation is employed. Special care must be taken to ensure the appropriate depth of the cannula to avoid postoperative complications. Secondly, for venous drainage in surgeries not requiring right atrium opening, cavoatrial cannulation is utilized. This is because separate superior and inferior vena cava cannulation can be challenging through left thoracotomy. During this process, it is crucial to dissect and ligate the azygos vein and dissect the phrenic nerve carefully to prevent injury (19). Thirdly, specialized surgical techniques involve several key points. These include proper manipulation of the ascending aortic cuff for enhanced visibility, careful insertion of the cavoatrial cannula with depth control, effective venting using carbon dioxide, correct placement of intracardiac defibrillation electrodes parallel to the heart's long axis, and the use of finer polypropylene sutures to accommodate the fragile tissue in pigs (19, 20).

Postoperative care plays a pivotal role in minimizing complications and enhancing survival rates in pigs. Due to the physiological differences between pigs and humans, specialized care is indispensable. It is advisable to promptly remove catheters during recovery, unless continuous monitoring is essential. In such cases, secure fixation devices and individual housing are crucial to prevent accidental detachment of arteriovenous lines (20). Maintaining fluid balance is another critical aspect, which involves continuous assessment of feeding, drinking, and urination patterns, with timely fluid supplementation as required. Antimicrobial therapy should be administered for at least seven days, along with regular wound care and disinfection to prevent dressing displacement. Intercostal incisions generally pose a lower infection risk compared to sternotomy incisions. This is not only because sternum splitting is avoided but also due to the abundant blood supply in the strong muscles of the lateral chest wall, which acts as an effective barrier against bacterial invasion, as demonstrated in previous infection—related studies in porcine models.

**Figure 3:**
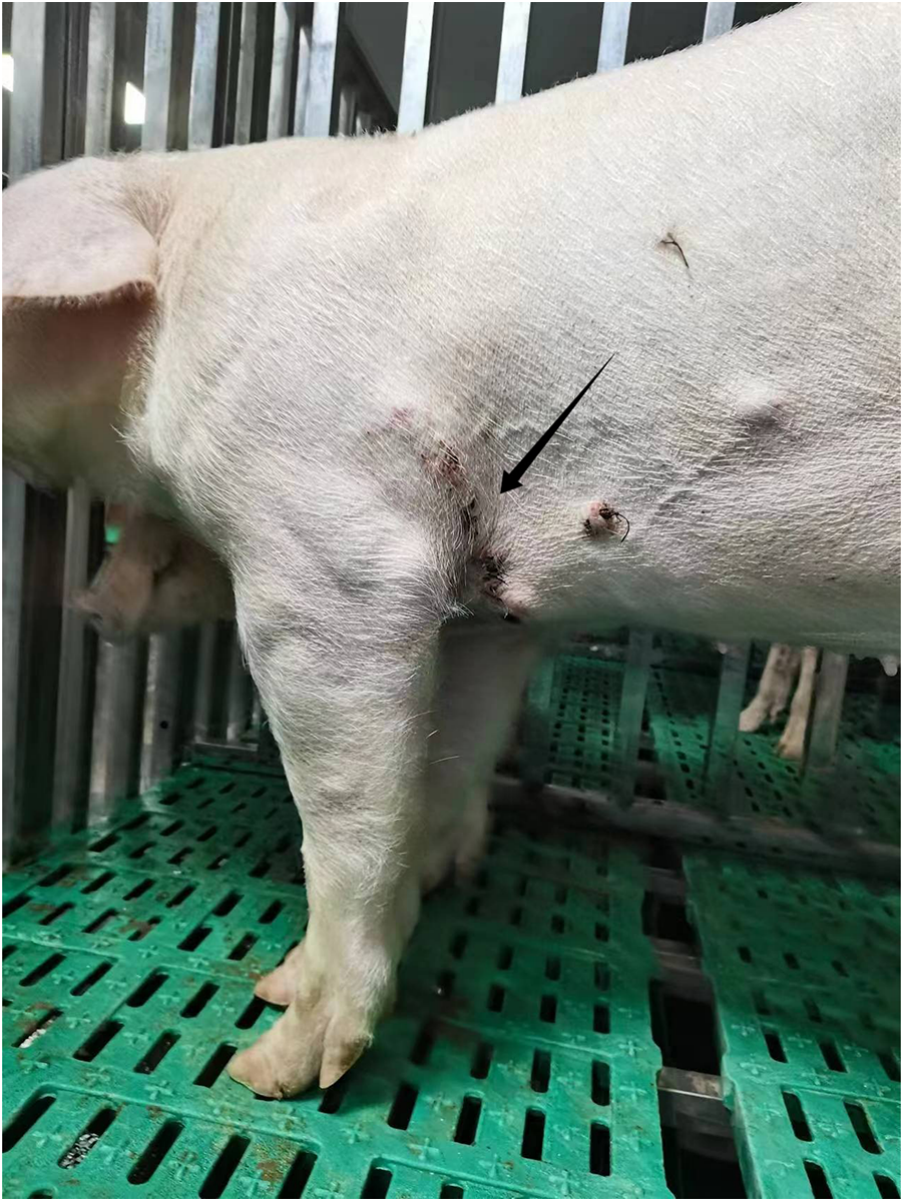
Displays a photograph of a pig that is standing and feeding itself on the second postoperative day. The arrow in the image highlights the condition of the postoperative incision site.

The low risk of nosocomial infection in pig housing, combined with the typically healthy state of experimental pigs, reduces the likelihood of complications such as septic shock. Close monitoring of drainage color and volume is essential, and any increase should be investigated immediately. Regular ultrasonographic or imaging examinations under anesthesia are also necessary. Moreover, maintaining pigpen hygiene is vital, involving prompt cleaning and waste removal to ensure wounds are not exposed to water (Figure 3).

Our retrospective study is based on data from several preclinical trials involving pigs, which were conducted for various new valve products. These pigs were euthanized upon reaching the post—operative follow—up period. So, the longest survival time we observed was 24 months. In fact, given their good physical condition, it is possible that the pigs could have survived for an even longer period.

While our study has achieved a three—year zero—mortality record, which can be attributed to standardized operational procedures and a highly skilled surgical team, it is not without limitations. The left thoracotomy approach, although advantageous for valve surgeries, may not be suitable for all cardiac procedures, highlighting the need for a tailored approach. The reproducibility of our results may be influenced by surgical expertise, which can vary among different research settings. Additionally, financial and space constraints associated with large animal models like pigs can pose challenges, potentially limiting the accessibility of this model for some research institutions. Prospective randomized controlled studies may be difficult to conduct in this context. However, due to differences in animal anatomy, the applicability of this surgical approach to other animal models still requires further exploration.

## 5 Conclusion

In conclusion, the surgical approach utilizing left thoracotomy has proven effective in significantly enhancing long—term survival rates in porcine heart surgeries. Moving forward, our ongoing efforts will focus on continuously improving surgical techniques to minimize postoperative complications, thereby enhancing the overall outcomes and applicability of this approach. Future research could potentially focus on investigating the influence of anatomical variations in different animals on the surgical outcome to delineate its broader application scope.

## Data Availability

The raw data supporting the conclusions of this article will be made available by the authors, without undue reservation.
